# Long‐term follow‐up in preschool children after radiofrequency catheter ablation of arrhythmias

**DOI:** 10.1002/joa3.12827

**Published:** 2023-03-10

**Authors:** Liliya I. Svintsova, Sergey N. Krivolapov, Olga Y. Dzhaffarova, Irina V. Plotnikova

**Affiliations:** ^1^ Cardiology Research Institute, Tomsk NRMC Tomsk Russia

**Keywords:** arrhythmia recurrences, children, complications, effectiveness, radiofrequency ablation

## Abstract

**Background:**

Radiofrequency ablation (RFA) is the standard method of treatment for tachyarrhythmias in school children, and it leads to complete recovery in children without structural heart disease. However, RFA in young children is limited by the risk of complications and unstudied remote effects of radiofrequency lesions.

**Objective:**

To present the experience of RFA of arrhythmias and the results of follow‐up of younger children.

**Materials and Methods:**

RFA procedures (*n* = 255) were performed in 209 children with arrhythmias from 0 to 7 years old. The arrhythmias were presented with atrioventricular reentry tachycardia with Wolff‐Parkinson‐White (WPW) syndrome (56%), atrial ectopic tachycardia (21.5%), atrioventricular nodal reentry tachycardia (4.8%), and ventricular arrhythmia (17.2%).

**Results:**

The overall effectiveness of RFA, considering the repeated procedures performed due to the primary ineffectiveness and recurrencies, was 94.7%. There was no mortality associated with RFA in patients, including young patients. All cases of “major” complications are associated with RFA of the left‐sided accessory pathway and tachycardia foci and are represented by the mitral valve damage in three patients (1.4%). Tachycardia and preexcitation recurred in 44 (21%) patients. There was a correlation between recurrences and parameters of RFA (odds ratio 0.894; 95% confidence interval: 0.804–0.994; *p* = .039). Reducing the maximum power of effective applications in our study increased the risk of recurrence.

**Conclusion:**

The use of the minimum effective parameters of RFA in children reduces the risk of complications, but increases arrhythmia recurrence rate.

## Graphical abstract

The use of the minimum effective parameters of RFA in small children reduces the risk of complications, but increases arrhythmia recurrence rate.

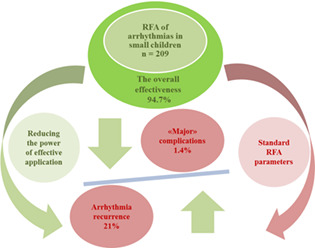

## 1 ACTUALITY

Radiofrequency ablation (RFA) for arrhythmias is a radical treatment for cardiac arrhythmias in children, regardless of age. Until now, experts continue to discuss the safety and long‐term efficacy of RFA in young children due to the high risk of complications of the procedure and unexplored long‐term results of interventional treatment.^1^, ^2^, ^3^ It should be noted that a fairly limited number of clinics in the world have experience in RFA for arrhythmias in this age group.

## 2 STUDY METHODS

### 2.1 Clinical characteristics of patients

**FIGURE 1:**
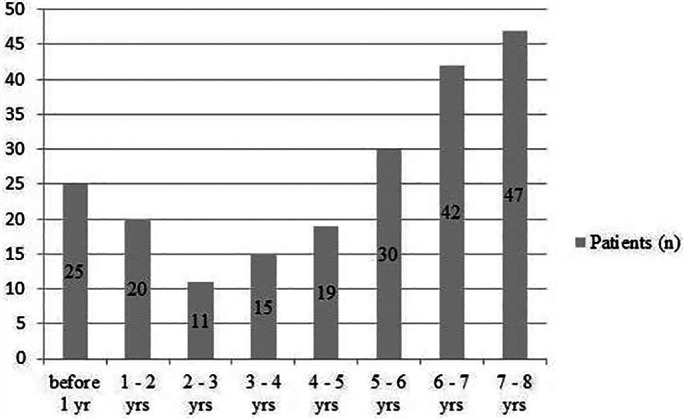
Age distribution.

A prospective, nonrandomized study was carried out between 2004 and 2018. RFA procedures (*n* = 255) were analyzed in 209 patients with arrhythmia aged 0–7 years, including 25 patients under 1 year of age. Inclusion criteria for the study were the absence of congenital heart disease, laboratory signs of myocarditis, and acute infectious diseases; the presence of paroxysmal, sustained and incessant tachycardias, ventricular extrasystole with high daily burden of 25% with episodes of unstable ventricular tachycardia; ineffective drug therapy and/or signs of reduced cardiac function. The patients' age distribution is shown in Figure 1. The average age at the time of RFA was 4.5 ± 2.2 years; the average weight was 19 ± 5.8 kg. Written informed consent was obtained from the parents of all patients before the intracardiac electrophysiological study (EPS) and RFA. The study was approved by an ethics committee (Biomedical Ethics Committee). Antiarrhythmic drugs were discontinued no later than five half‐lives before the procedure.

Clinical and electrophysiological characteristics and mechanisms of arrhythmia are presented in Table 1. Supraventricular tachycardias (SVT, *n* = 173) including atrioventricular reentrant tachycardias (AVRT) with Wolff‐Parkinson‐White (WPW) syndrome were in 118 patients (56%), ectopic atrial tachycardias (EAT)—in 45 patients (21.5%), and atrioventricular nodal reentrant tachycardia (AVNRT)—in 10 patients (4.8%). Ventricular arrhythmias (VT) occurred in 36 patients (17.2%) and were presented by ventricular extrasystole with high daily burden 25% with episodes of unstable ventricular tachycardia.

**TABLE 1:** Clinical and electrophysiological characteristics of patients and mechanisms of arrhythmia.

Mean age (*М* ± SD) (years)	4.5 ± 2.3
Mean weight (*М* ± SD) (kg)	19.0 ± 5.8
Sex: boys/girls	116/93
Cardiac dysfunction signs, *n* (%)	108 (51.7%)
Functional class II–IV (NYHA), *n* (%)	43 (20.6%)
WPW syndrome, *n* (%)	118 (56%)
Manifest/concealed (*n*)	90/28
Right‐sided accessory pathway/including paraseptal (*n*)	71/34
Left‐sided accessory pathway/including paraseptal (*n*)	47/4
Atrial tachycardia, *n* (%)	45 (21.5%)
Right atrial tachycardias/including parahisial (n)	39/16
Left atrial tachycardias (*n*)	6
AV‐nodal reentry tachycardias, *n* (%)	10 (4.8%)
Ventricular arrhythmias, *n* (%)	36 (17.2%)
Right ventricular (*n*)	25
Left ventricular (*n*)	11

Abbreviation: SD, standard deviation, WPW, Wolff‐Parkinson‐White, NYHA, New York Heart Association.

Paroxysmal course of tachycardia was observed in 118 patients (68% of all SVT). Most of such tachycardias were presented by AVRT with WPW syndrome and AVNRT. Sustained tachycardia occurred in patients with atrial ectopic tachycardia (*n* = 47.3%). Incessant atrial ectopic tachycardia was observed in eight patients (5%) at the age of 1 year old.^4^

Heart failure signs were observed in 43 patients (20.6%), of which functional class (FK) IV—in 2 patients, III FK—in 13 patients, and II FK—in 28 patients (Table 1).

Median values of cardiac function in study population were as follows: left ventricle ejection fraction (LV EF)—65.0% (interquartile range [IQR] 52.0–70.0), LV end‐diastolic volume (EDV) (% of normal)—116.0% (IQR 96.5–135.5), left atrium (LA) volume—121.0% (IQR 96.9–159.3), and right atrium (RA) volume—120.0% (IQR 99.5–146.0).

There were 108 (51.7%) cases of cardiac dysfunction. In 48 (23%) patients, there was reduced EF. Decreased LV EF considered as <55%, EF was assessed by Simpson. Twenty percent of patients had increased LV EDV > 120% of normal, 38% had increased LA volume >130% of normal, and 35% had increased RA volume >130% of normal.

### 2.2 Technology of intracardiac EPS and RFA

Intracardiac EPS and RFA were performed with well‐balanced total intravenous anesthesia that provided by continuous infusion of propofol and fentanyl. Ventilation was used during RFA in patients till 1 year old. According to Seldinger technique, right and left femoral veins through which diagnostic and ablation catheters passed were punctured. Positioning and manipulation of the catheters was performed under X‐ray control. A 5Fr catheter was used in patients under 20 kg and 7Fr—in patients weighed more than 20 kg. During electrophysiological studies, the catheters were placed in the upper area of the RA and the right ventricle to pace and record electrograms. The examined group of children was limited to two inserted catheters—a diagnostic Viking Boston Scientific 5Fr and ablation Marinr™ 5Fr Medtronic (SCXS and SC). RFA with 3D mapping system CARTO Biosense Webster with NaviStar 7Fr catheters was performed in patients with atrial ectopic tachycardia. Navigation mapping was used in children weighing less than 15 kg after a preliminary ultrasound assessment of the diameter of the femoral veins. A transesophageal diagnostic catheter was placed to record the atrial signal and pace the atria in case when it was necessary to use a third reference catheter, or if it was impossible to puncture one of the femoral veins.

After arrhythmia mapping, radiofrequency applications were performed starting from the temperature of 50°C and the power from 10 to 20 W during 10 s. If the ablation was successful, the applications continued up to 30 s. If the arrhythmia restored, maximum temperature and power raised to 55°C and 30 W, consequently.

If the tachycardia substrate was localized in the left heart, in the absence of a persistent foramen ovale (PFO) in the patient, a retroaortic approach was used through the femoral artery or transseptal puncture according to the standard technique under the control of transthoracic echocardiography (Echo). PFO was used in 14 patients. In 26 patients, the access to the left heart was performed retrogradely through the femoral artery. Transseptal puncture was performed in six patients with a high risk of femoral vein catheterization due to its insufficient diameter.

Heparin 50 units/kg therapy with further intake of low‐dose aspirin during 3 weeks for thromboembolic complications prophylaxis was used at RFA of left‐sided arrhythmia substrates.

### 2.3 Postoperative observation and follow‐up

All patients after RFA were observed in an intensive care unit with continuous electrocardiographic monitoring during 24 h. The first control examination including electrocardiogram (ECG), Holter monitoring, and Echo was performed in 5–7 days after RFA and then in 1, 3, 6, and 12 months. The follow‐up was 5.7 ± 2.9 years (from 1.5 to 10 years).

### 2.4 Statistical analysis

Statistical analysis of the given data was carried out using STATISTICA 6.0 for Windows. The description of quantitative characteristics, the distribution of which did not correspond to the normal law, is presented in the form of a median (Me) and IQR. If the quantitative characteristics had normal distribution, their description is presented in the form of (*M* ± σ). The rating description was performed by the contingency table with absolute and relative frequency (%) of characteristics. To determine statistically significant differences in nominal characteristics, the analysis of contingency table was used (Pearson *χ* ^2^ criteria, two‐sided Fisher exact test). The comparison of two independent samples was performed by Mann–Whitney test. *p* < .05 was considered as statistically significant difference. The calculation of the probability of realization of an unfavorable prognosis and identification of significant predictors of an unfavorable outcome were carried out by the multivariate logistic regression analysis with direct stepwise inclusion of variables into the model.

## 3 STUDY RESULTS

### 3.1 Indications for RFA

Symptomatic arrhythmia, reduced cardiac function, heart failure signs, and refractoriness to antiarrhythmic therapy (AAT) are the indications for RFA.^5^ The choice of ablation method of arrhythmia treatment by the parents in case of their refusal to get preventive AAT and limit children in sports was the indication for RFA in six children aged 5–7 years. WPW syndrome occurred in all these patients.

### 3.2 Previous AAT

AAT as the first stage of the treatment was used in all patients. Antiarrhythmic drugs were prescribed by a selective method: If one drug was ineffective, another was prescribed.^6^ In average, each patient got three drugs consecutively (from 1 to 5). The duration of therapy with each drug ranged from 2 weeks to 3 months.

With ineffective monotherapy, the combined therapy was prescribed: amiodarone + propranolol, amiodarone + digoxin, and propafenone + propranolol. AAT in the given patient group was ineffective or had only a temporary effect consisting in decrease of heart rate (HR) during first days of tachycardia treatment. Later, HR reached the initial tachycardia level, and cardiac dysfunction continued to progress that was the indication for RFA.

### 3.3 Efficacy and complications of RFA

The overall efficacy of RFA in all patients including repeated procedures performed due to ineffective primary RFA and recurrences was 94.7%. RFA efficacy in children under 1 year of age was 96%.

There was no mortality associated with RFA.

During RFA, injury of the mitral valve (MV), considered as a “major” complication, occurred in three patients with left‐sided accessory pathway (1.4%; 3/209), among which the age of one patient was 6 months and two—7 years. At the age of 4, the first patient required MV repair in 3.5 years after RFA due to III grade mitral regurgitation and heart failure. MV revision discovered two clefts—of anterior leaflet in A1 segment and of posterior leaflet in P1 segment. That corresponds to sites of RFA applications. There was II grade mitral regurgitation during follow‐up in the 2^d^ and 3^d^ cases, without heart failure signs. There were no other severe “major” complications of RFA (complete atrioventricular block, cardiac perforation, and coronary artery injuries) in study population.

Transitory, or so‐called “minor,” complications were in 28 (13.4%) of patients. They were presented by transient AV blocks of 1–3°, nodal rhythm, and His bundle block associated with catheter manipulations during intracardiac EPS and RFA. It should be noted that 15 patients (53.6%) of 28 children having transient AV block, nodal rhythm, and His bundle block in the EP laboratory had paraseptal localization of accessory pathway, parahisian localization of atrial ectopic foci. One patient had AVNRT. It is known that RFA of arrhythmias with these localizations of accessory pathways and arrhythmia foci is associated with a risk of AV block.^5^ The association of transient AV blocks and His bundle branch blocks with RFA of tachycardias localized in the area of the AV node and bundle of His was statistically significant (*p* < .001). According to ECG and Holter monitoring during follow‐up, AV conduction disorders were not observed in all patients with intraoperative transient AV block.

### 3.4 Arrhythmia recurrences

Tachycardia and preexcitation recurred in 44 (21%) patients. In 20 children, the recurrences occurred in the first 5 days after RFA, in 24—from 5 days to 6 months. Clinical characteristics of patients with recurrences are presented in Table 2. Repeated RFA was performed in 40 patients. Children after ineffective repeated RFA (*n* = 2) and with repeated recurrences (*n* = 4) required additional RFA. The overall effectiveness considering recurrences and repeated RFA was 94.7%. The period of recurrence after primary RFA was 0.5 months (IQR 0.1–2.0)—from 1 day to 6 months. The interval between primary and repeated RFA was 6.0 (IQR 3.0–17.0) months. The interval between the second and third procedures in six patients ranged from 2 weeks to 20 months. In patients with recurrences, atrial and ventricular tachycardia prevailed.

**TABLE 2:** Clinical characteristics of patients with arrhythmia recurrences after RFA.

Recurrence (total/immediate/remote)	44/20/24
Repeated RFA/additional RFA	40/6
Sex (m/f)	29/15
The time of recurrence after primary RFA, months (Ме(IQR))	0.5 (0.1–2.0)
The interval between primary and repeated RFA, months (Me (IQR))	6.0 (3.0–17.0)
Recurrences in children with WPW syndrome (*n*; % in the given arrhythmia group)	21; 18%
Recurrences in children with atrial tachycardia (*n*; % in the given arrhythmia group)	12; 27%
Recurrences in children with AVNRT (*n*; % in the given arrhythmia group)	1; 10%
Recurrences in children with ventricular tachycardia (*n*; % in the given arrhythmia group)	10; 28%

Abbreviations: AVNRT, atrioventricular nodal reentrant tachycardia; IQR, interquartile range; RFA, radiofrequency ablation.

We compared patients with and without recurrences to assess potential predictors of arrhythmia recurrence after successful RFA. Age, type of arrhythmia, parameters of radiofrequency exposure, and localization of the arrhythmogenic zone were considered as possible predictors of recurrences. There was no statistical significance in patients with and without recurrences of different age groups (*F* 2.431; *p* = .251) and different types of arrhythmias (AVRT, AVNRT, EAT, and VT) (*F* 1.880; *p* = .785). Right‐ or left‐sided localization of arrhythmia focus or accessory pathways in patients with SVT had no significant effect on the occurrence of recurrences (*χ* ^2^ 1.438, *p* = .231). Among parameters of RF exposure, only the maximum power of effective applications had statistically significant differences in patients with (22.5 W; IQR 20.0–30.0) and without recurrences (30.0 W; IQR 25.0–40.0) (*p* = .003) (Table 3). According to logistic regression analysis, a relationship between recurrences and radiofrequency exposure parameters was established (odds ratio 0.894; 95% confidence interval: 0.804–0.994; *p* = .039).

**TABLE 3:** Parameters of RF exposure in patients with and without recurrences.

Parameters of radiofrequency exposure	Patients without recurrences	Patients with recurrences	*p*
Procedure time, min, (Me(IQR))	120 (60–165)	115 (90–120)	.597
Fluoroscopy time, min, (Me(IQR))	18.5 (10–35)	18 (10–28)	.477
Max. temperature of successful applications, °C, (Me(IQR))	60 (55–60)	57.5 (55–60)	.350
Max. power of successful applications, W, (Me(IQR))	30 (25–40)	22.5 (20–30)	.003

Abbreviations: IQR, interquartile range; RF, radiofrequency.

## 4 DISCUSSION

This study presents our experience of RFA of arrhythmias in 209 younger children from 0 to 7 years. The overall effectiveness of RFA, considering arrhythmia recurrences after initially successful RFA and repeated procedures, was 94.7%. There was no mortality associated with the RFA procedure. “Major” complications, amounted to 1.4%, were associated with left‐sided localization of the arrhythmia substrate and represented by damage of the MV. “Minor” or transient complications were mostly transient AV block of 1–3° (14.4%). Recurrence of arrhythmias after primary RFA was observed in 21% of patients.

Limited number of clinics worldwide has experience of RFA in children of the first years of life.^5^, ^8^, ^9^

Table 4 presents the results of RFA in small patients according to data from various arrhythmological centers all over the world.^2^, ^10^, ^11^, ^12^, ^13^, ^14^, ^15^, ^16^, ^17^

**TABLE 4:** Results of catheter ablations in younger children according to various arrhythmology centers data.

Publication author	Publication year	*n*	Age/body weight	Efficacy (%)	Complications		Recurrences
					Major	Transitory	
Aiyagari et al.	2005	25	2.8 ± 1.9 years	91	8%	0	4%
Chiu et al.	2009	27	3.9 ± 1.7 years	93	3.7%	3.7%	7.4%
Tumer et al.	2011	17	0–2 years	88	0	17.6%	23.5%
Melo et al.	2012	18	3.0 ± 1.9 years	79	0	12%	17%
An et al.	2013	25	0–9 years	97.9	‐	‐	11.6%
Backhoff et al.	2016	22	0–3 years	82	9%	4.5%	‐
Jiang et al.	2016	123	2.3 ± 0.8 years	94.5	‐	‐	6.8%
Ozaki et al.	2018	22	1.0 ± 0.6 years	90.9	3.8%	3.8%	15%
Kato et al.	2019	104	<15 kg	85.6	0.9%	‐	29.2%

“Major” complications in Aiyagari et al., Chiu et al., and Kantoch et al. are represented by complete AV block, required the implantation of a permanent pacemaker, and atrial perforation, which in half of the cases resulted from transseptal puncture.^1^, ^10^, ^11^ According to the PAPCA (Prospective Assessment After Pediatric Cardiac Ablation) study, which presents prospective results of patients after catheter ablation according to the pediatric RFA registry, the risk of complete AV block is 1%–2% with anterior septal, and up to 3%—with midseptal localization of the tachycardia substrate.^7^ Such “major” complications were avoided in our study. They also were not observed in patients with transient intraoperative AV conduction disorders during follow‐up.

In our study, “major” complications were associated with left‐sided localization of arrhythmias and catheter manipulations in the MV. The technology and equipment require improvement considering the need to accompany the RFA procedure with imaging methods, since such complications are usually disclosed after RFA. Intracardiac echocardiography is used during RFA in adult invasive arrhythmology. However, in pediatric population with weight less than 30 kg, the use of the technique is limited by the necessity of additional venous access and catheter size 9Fr.^18^ Clark et al. present the experience of transesophageal echocardiography for RFA of left‐sided accessory pathway. Still, its use is limited by the need for additional equipment for electrophysiological laboratories and has not yet become widespread, especially in pediatric electrophysiology.^19^ Hence, transthoracic echocardiography is necessary in this category of patients as a control during RFA of left‐sided arrhythmia foci and accessory pathway.

Recurrences make the greatest contribution to the structure of long‐term results after RFA of arrhythmias. Several follow‐ups in pediatric population show that the recurrences after primary successful RFA occurred in 4.9%–29.2%.^7^, ^10^, ^11^, ^12^, ^13^, ^14^, ^15^, ^16^, ^17^ The period of recurrence after primary RFA was 0.5 months (IQR 0.1–2.0) ‐ from 1 day to 6 months in our study. Our data confirm the reports of other authors that the maximum period of recurrences does not exceed 6 months, and only in single cases recurrences occur in a longer period.^7^, ^11^ Despite high efficiency of primary RFA of arrhythmias in children of early age, there are still many controversial issues concerning the causes of arrhythmias recurrences during follow‐up. The features of the anatomical location of the tachycardia substrate, multiple accessory pathways or ectopic foci, and the inaccuracy of arrhythmia mapping were considered as the causes of recurrences.^7^

The comparison of our results with previous studies with RFA outcomes in younger children allows us to conclude that along with the minimum number of “major” complications, we got a sufficiently high recurrence rate that required repeated procedures and ensured a high overall RFA efficacy in our patients. It is well known that the risk of injury and perforation of myocardium is inversely related to the age and weight of the patient. The factors reducing the risk of catheter treatment of arrhythmias in children are the experience of the electrophysiologist associated with delicate catheter manipulation in small children due to thin walls and small chambers of the heart and minimally effective parameters of radiofrequency exposure.

## 5 CONCLUSION

The most serious “major” complications are associated with the left‐sided localization of the focus of arrhythmia or accessory pathway. Therefore, the choice of management of such patients should be carried out considering the “risk” and “benefit” factors of RFA in each particular case, even if the clinic has considerable experience. The use of echocardiography during RFA of left‐sided arrhythmias and accessory pathway, especially in small patients, helps to reduce the risk of damage of valvular structures in radiofrequency exposure.

The association of recurrences with parameters of radiofrequency exposure identified because of regression analysis is related to the tendency of pediatric electrophysiologists to use minimally effective parameters of radiofrequency exposure, such as temperature, power, and application number to decrease the risk of complications.

The use of minimally effective RF exposure parameters in children reduces the risk of complications, but increases the possibility of arrhythmia recurrences.

## AUTHOR CONTRIBUTIONS

Liliya I. Svintsova contributed to development of the article concept, analysis and interpretation of results, direct research of the rhythm disturbances included in the section, and analysis of the data obtained and participated in writing the text of the article. Sergey N. Krivolapov was involved in performing intracardiac electrophysiological study and radiofrequency ablation and participated in the manuscript writing. Olga Y. Dzhaffarova contributed to participation in analysis and interpretation of the results, patient consultations, conducting noninvasive research methods included in the rhythm disturbances section, and analysis of the data obtained. Irina V. Plotnikova contributed to interpretation of the results and participation in writing the text of the article.

## CONFLICT OF INTEREST STATEMENT

Authors declare no conflict of interests for this article.

## CONSENT APPROVAL

The authors confirm that written consent for submission and publication of this paper has been obtained from the patient in line with COPE guidance.

## DECLARATIONS

*Approval of the research protocol*: No human participant was involved in this study. *Registry and the registration*: N/A. *Animal studies*: N/A.
